# Attentional reorganization in heritage environments: depth of engagement and the expression of environmental decline

**DOI:** 10.3389/fpsyg.2026.1800968

**Published:** 2026-10-20

**Authors:** Shengchao Li, Qing Xia, Shiying Huang, Sining Huang, Yingyuan Lv, Yuhui Jiang, Xiaoling Chen

**Affiliations:** School of Management, Guangdong University of Education, Guangzhou, China

**Keywords:** embodied cognition, environmental perception, explainable AI (XAI), place attachment, recreation specialization, solastalgia, user-generated content

## Abstract

**Introduction:**

Whether deeper engagement with a heritage place shields visitors from its environmental decline or sensitizes them to it remains unclear. This study examines how depth of engagement is associated with the way environmental conditions enter visitors' accounts of Xiaozhou Village, an artist-settled urban village in Guangzhou, China.

**Methods:**

We assembled 12,493 posts and comments from 13 platforms, annotated sentiment at the record level, fine-tuned a Chinese transformer classifier, coded every post for depth of engagement and for the author's position toward the village, built 18 transparent lexicon features describing its spatial elements, and used Shapley additive explanations to attribute the classifier's predictions to those features.

**Results:**

The share of negative accounts rises monotonically with depth of engagement: 10.7% among those passing through, 22.8% among those describing a dedicated visit and 28.6% among those who return repeatedly or stay. The contrast survives adjustment: with the amount each author writes, the platform, the type of activity and the author's position toward the village all controlled, the odds of negative expression among those who return repeatedly or stay are 2.14 times those of passers-by, while length of post carries no independent association; the contrast at the intermediate level is smaller and depends on who is writing. Nor does the gradient reduce to a single activity: sketching shows no detectable association with negative expression, and photography is associated with markedly less of it. Residents are the most critical group and commercial accounts the least.

**Discussion:**

These findings support a situated-cognition account in which perceived environmental quality emerges not from objective conditions alone but from practice-mediated attentional organization, and they specify what conditions that organization: the depth and position of a visitor's involvement rather than the kind of activity pursued. The design is cross-sectional and observational, and all findings are stated as associations. The study extends recreation specialization theory into heritage tourism and identifies solastalgia among those most deeply involved in a changing place.

## 1 Introduction

What happens when a centuries-old heritage village becomes an Instagram sensation? Xiaozhou Village in Guangzhou presents a paradox. While heritage scholars document visible degradation—polluted waterways, commercialization—millions of tourists flood its narrow alleys seeking “authentic” Lingnan culture. This disjuncture between material condition and experiential value is, at bottom, a question about attention: what a visitor registers of a place, what recedes from view, and what determines the difference. It is therefore a question for environmental psychology: how attention to a setting is organized.

This is not a simple tale of heritage loss, nor one of successful preservation. Xiaozhou exemplifies the pattern this paper sets out to explain: living heritage sites that sustain, and sometimes amplify, perceived authenticity while their material fabric fragments. Unlike Europe's restored medieval towns or UNESCO World Heritage Sites, Xiaozhou operates through informality: unregulated artistic interventions, spontaneous commercial adaptations, and fluid boundaries between “heritage zone” and “urban village” (Ling and Wang, 2024). Its vitality emerges not from static preservation but from continuous reconfiguration—what Deleuze and Guattari term “assemblage” (Deleuze and Guattari, 1988).

The phenomenon unsettles a foundational assumption in heritage tourism scholarship: how can a site maintain, and potentially intensify, its perceived cultural authenticity while undergoing visible physical decay? What psychological mechanisms organize a visitor's attention such that some features of a deteriorating site come to the fore and others recede—and does deeper involvement with a place strengthen that filtering or dismantle it? Environmental psychology offers competing answers: accounts built on processing fluency and effort justification predict that involvement makes evaluation more favorable, while accounts built on recreation specialization and solastalgia predict the reverse. The answer also bears on governance where conventional preservation paradigms have little purchase.

Heritage tourism scholarship faces three interconnected limitations here. The first is the persistence of static preservation paradigms: recent literature acknowledges heritage as “process” (Harrison, 2012) or “practice” (Smith, 2006), yet analytical frameworks remain anchored in conservation logic, measuring “authenticity loss” against historical baselines and framing success as minimizing change (Timothy and Nyaupane, 2009); even critical heritage studies problematizing Authorized Heritage Discourse default to preservation as the normative horizon (Meskell, 2015). In practice, many living heritage sites, informal settlements above all, thrive through reconfiguration, sustaining cultural practice through continuous assemblage and disassembly (Volo and Wegerer, 2023; Huang and Xu, 2025; Zhang et al., 2025). What requires explanation is not how to freeze such sites but how assemblage generates stability of experience despite variance in fabric.

The second is a flat spatial epistemology. Spatial analysis in heritage tourism predominantly employs Euclidean metrics: visitor flows map as linear movements, hotspots emerge through density calculations, and “sense of place” derives from architectural clustering (Shoval and Ahas, 2016). Geographic Information Systems reinforce this ontology, in which meaning follows from geometric proximity. Recent work in cultural geography, drawing on Deleuze and topology, suggests instead that experience operates through folded space (Deleuze, 1993): elements achieve psychological nearness through affective intensity rather than metric distance (Jia et al., 2024; Zhu and Xu, 2024; Lapointe, 2025), so that negative environmental cues—pollution, noise, commercial clutter—may be psychologically folded away even as they pervade physical geography.

Existing heritage studies lack conceptual tools for analyzing such dynamics. Topological vocabulary has begun to enter heritage tourism research: Tang and Li (2026) analyze intangible heritage in a Guangzhou urban village through cycles of folding and unfolding, showing how cultural practice persists through spatial transformation. That account is ethnographic, and it treats folding as a property of the heritage space itself. What remains undeveloped is a treatment in which the fold is a property of the visitor's engagement rather than of the site, and in which its direction can be estimated rather than interpreted.

The third is the interpretability of computational tourism research. User-generated content has enabled tourism research at a scale not previously available, yet the “black box problem” limits its theoretical contribution (Solakis et al., 2024; Wang et al., 2025; Zhou et al., 2025). Tree-based models predict satisfaction from review text with useful accuracy, but the explanatory mechanisms remain opaque—and for heritage research it is mechanisms that are at issue: knowing that artistic activity correlates with positive sentiment helps little if one cannot say through what process art works. Explainable AI, particularly SHAP (SHapley Additive exPlanations), quantifies each feature's marginal contribution to predictions, but tourism scholarship has treated it mainly as technical validation rather than as a method for generating substantive findings.

This study addresses the identified gaps through a case analysis of Xiaozhou Village, guided by three interrelated questions grounded in environmental psychology. RQ1. How do visitors reorganize environmental attention when engaging with a heritage site? (Processing Fluency Theory: Reber et al., 2004.) RQ2. Do positive experiences attenuate negative environmental cues, or does deeper involvement heighten sensitivity to them? (Cognitive Dissonance Theory leaves this open in both directions: Festinger, 1957.) RQ3. What conditions that direction—the kind of activity a visitor pursues, or the depth at which they pursue it? We take these to be separable questions and test them separately, drawing on embodied and situated accounts of cognition (Varela et al., 1991; Wilson, 2002). Attention Restoration Theory (Kaplan, 1995) enters only for its premise that directed attention is a finite resource.

Four strands of the argument developed below bear on environmental psychology and on broader debates about human-environment interaction. The first adapts assemblage theory—prominent in urban studies (McFarlane, 2011) and political geography (Anderson et al., 2012) but underutilized in tourism research—for the analysis of living heritage. Unlike systems theory, which presumes functional integration toward equilibrium, assemblage thinking treats heritage sites as provisional configurations of heterogeneous elements related through contingent coding and territorialization (Volo and Wegerer, 2023; DeLanda, 2016); rather than asking what attributes make Xiaozhou authentic, which presumes authenticity as an inherent property, we ask how assemblage processes generate authenticity effects.

The second develops topological folding as an affective-spatial mechanism (Lury et al., 2012; Martin and Secor, 2014; Tang and Li, 2026): the way visitors psychologically redistribute attention in experiential space through affective intensities that reorganize spatial proximities (Massumi, 2021; Zhu and Xu, 2024; Lapointe, 2025). The term is needed because cognitive filtering and selective perception presuppose a single direction, whereas here the direction is itself the variable; and where Tang and Li track folding as something a heritage space undergoes, we ask what the fold looks like from the position of the visitor. Our analysis indicates that the fold is not unidirectional: whether low-resonance elements such as pollution, noise and commercial clutter recede to topological distance depends on how deeply the practice is invested. This is neither denial nor ignorance—degradation is named explicitly in a substantial share of posts, and what differs systematically is the weight it carries. It challenges MacCannell's “staged authenticity,” which assumes tourists either penetrate to a back stage or remain duped by a front stage (MacCannell, 1973); Xiaozhou's visitors do neither.

The third concerns what SHAP can do methodologically: by quantifying each spatial feature's marginal contribution to sentiment predictions it serves to locate substantive patterns rather than merely to validate a model, though what it uncovers are non-linear associations and feature importances that traditional regression often misses, not causality in the experimental sense (Li et al., 2024; Zhou et al., 2025).

The fourth concerns governance for informal heritage in the Global South, where China's urban villages, India's unauthorized colonies, Southeast Asia's kampungs and Latin America's favelas lack formal conservation frameworks yet sustain cultural practice through vernacular adaptation (Simone, 2004; Roy, 2005), and where conventional preservation logic, premised on boundary delineation and material stabilization, proves inapplicable (Huang and Xu, 2025; Zhang, 2025). Rather than attempting to freeze heritage assemblages, policy might nurture the conditions enabling continuous reconfiguration: protecting not specific buildings but capacities for assemblage—spaces for artistic practice, platforms for community ritual, infrastructures supporting affective engagement. For the informal settlements across Asia, Africa and Latin America in which more than a billion people now live (UN-Habitat, 2024), this implies a shift from heritage-as-monument to heritage-as-process. Figure 1 sets out the resulting framework.

**Figure 1:**
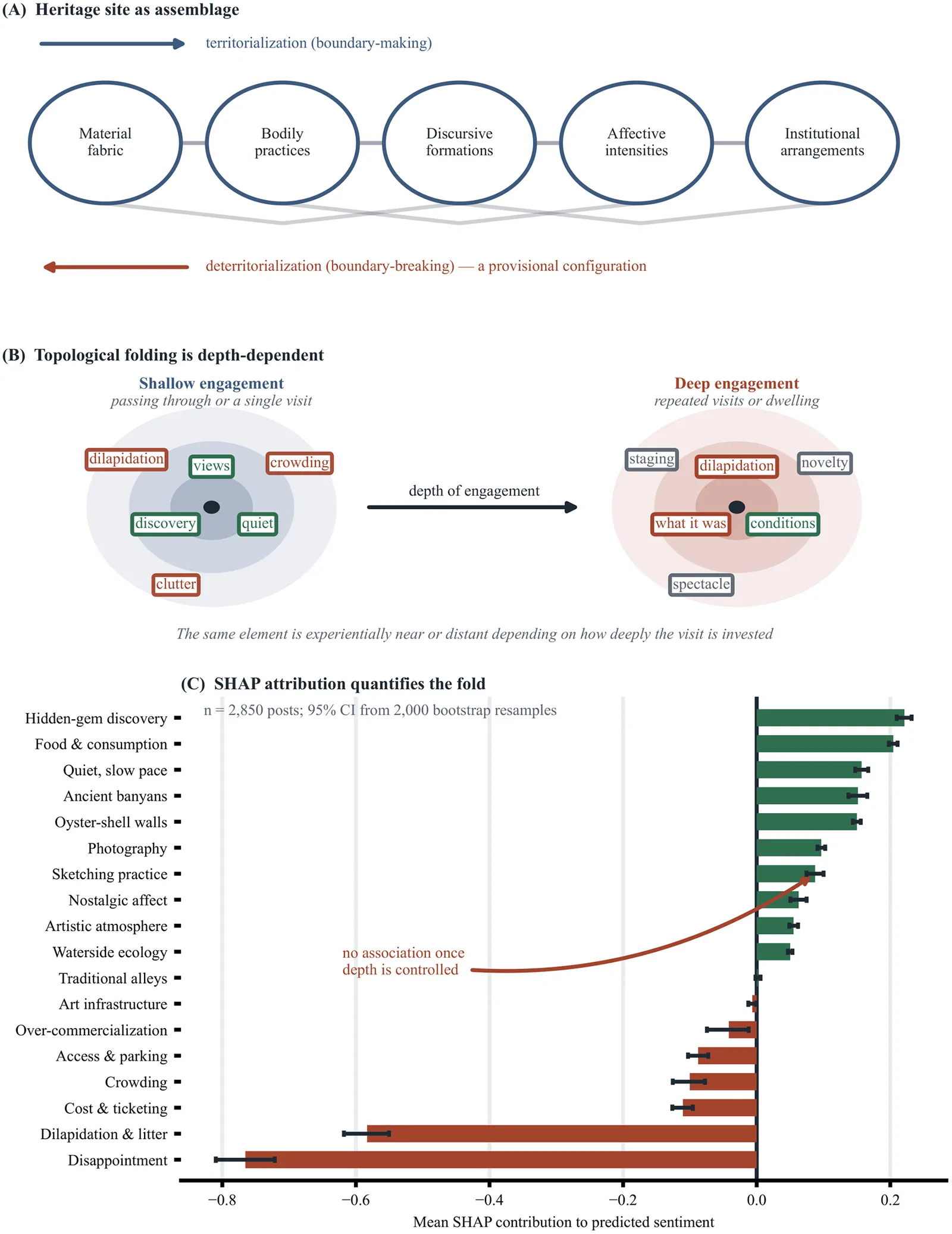
Theoretical framework: heritage assemblage, topological folding and explainable attribution. **(A)** represents a heritage site as a provisional assemblage of material, social, affective and discursive elements held together by continuous territorialization and deterritorialization. **(B)** represents the topological space in which those elements acquire variable affective intensity, so that the same element may be experientially near or distant depending on the depth of a visitor's engagement. **(C)** is not schematic: it reports the estimated mean SHAP attribution of each lexicon feature, with 95% confidence intervals from 2,000 bootstrap resamples (n = 2,850 posts).

## 2 Literature review

### 2.1 From monument preservation to living heritage assemblages

Recognition of these disjunctures catalyzed a paradigm shift toward “living heritage”—heritage understood as practice and social process rather than static artifact—institutionally acknowledged in UNESCO's 2003 Convention for the Safeguarding of Intangible Cultural Heritage (UNESCO, 2003). Yet as Meskell notes, documentation and codification often render intangible heritage tangible, transforming fluid practices into frozen traditions (Meskell, 2015). Because authenticity recurs throughout the analysis that follows, we state at the outset the sense in which we use it: not as a property residing in material fabric and verifiable against a historical baseline, but, following the constructivist and performative turn in heritage and tourism studies, as an effect produced relationally and continually renegotiated—something a place is granted in the course of being visited, worked in and written about. This carries a consequence that matters for what follows: if authenticity is produced through engagement, then different kinds of engagement may produce opposed judgments about the same place. The analysis reported below finds exactly that.

Scholarship on informal settlements and vernacular urbanism pushes this further. Herzfeld's Thai squatter communities sustain cultural belonging not by preserving buildings but through ongoing social practice (Herzfeld, 2016); Roy argues that informality is not a sector but a mode of urbanization whose flexibility and negotiability formal planning tends to extinguish (Roy, 2005); and Simone's “people as infrastructure” locates heritage value in relational networks rather than material substrates (Simone, 2004). China's urban villages (chengzhongcun) exemplify the type (Zhang et al., 2025): encircled by urban expansion yet retaining rural governance structures, they host ancestral halls alongside karaoke bars, Hakka dialects amid migrant laborers, and traditional festivals interwoven with e-commerce logistics. Debate has centered on their precarity rather than their vitality (Zhang et al., 2025). How they sustain cultural distinctiveness amid homogenizing forces is a question that cannot be answered within preservation-vs.-development binaries.

### 2.2 Spatial production theory and topological relations

Topological accounts have so far treated folding as though it operated uniformly across visitors, yet the recreation literature suggests otherwise. Bryan arrays participants in a single activity along a continuum from the general to the specialized (Bryan, 1977), and more specialized participants have repeatedly been found to hold more exacting standards for the resource itself, registering deficiencies that casual participants pass over; place attachment research points the same way from another direction, though to our knowledge it has not tested the possibility directly (Williams and Vaske, 2003).

### 2.3 The affective turn in tourism studies

Nor is affect in heritage settings only a matter of intensification. Where involvement is sustained rather than episodic, transformation registers as loss—the condition Albrecht and colleagues name solastalgia, the distress of those who remain in the place that is changing, sharpened by the sense of having no purchase on what is underway (Albrecht et al., 2007). A visitor arriving once encounters an atmosphere; someone who has painted in the same lanes across several years encounters a comparison.

### 2.4 Attention, attachment and the psychology of environmental perception

The questions this study asks are, in the end, questions about attention: what a visitor notices in a place, what recedes, and what conditions the difference. Environmental psychology offers three well-developed answers, and the study is organized around the tension between them and a fourth possibility. Processing Fluency Theory holds that stimuli which are easier to process are evaluated more favorably (Reber et al., 2004), implying that a setting offering ready-made compositions and recognizable scenes will be judged well largely irrespective of its condition. Cognitive Dissonance Theory predicts that visitors who have invested effort in a place will revise their evaluation upward through effort justification, so that the most committed should be the least critical (Festinger, 1957). Attention Restoration Theory supplies the premise underlying both: directed attention is a finite resource, and settings differ in how they draw on it (Kaplan, 1995).

These accounts converge on a single expectation—deeper engagement should mean more favorable evaluation—and it is that expectation this paper tests. The place attachment literature suggests it may be wrong. Place dependence, the functional bond formed through goal-directed activity, and place identity, the emotional and symbolic bond through which a place enters one's self-definition, are ordinarily treated as sources of favorable evaluation (Williams and Vaske, 2003); but an attachment strong enough to supply a standard against which a place is judged, and a stake in whether it meets that standard, might equally supply the grounds for criticism. Heritage settings put the two predictions in direct competition, because they draw both the briefest and the deepest forms of engagement to the same square meters. That competition is what this study is designed to adjudicate, and it is why the analysis below separates depth of engagement from type of activity: the fluency account predicts that what matters is the kind of encounter a visitor has, whereas the specialization and solastalgia accounts predict that what matters is how much of themselves they have put into the place.

### 2.5 Computational methods in tourism research

User-generated content from social media has enabled tourism research at a scale not previously available, yet computational approaches face the black-box problem: models predict outcomes without explaining mechanisms (Lundberg and Lee, 2017; Lipton, 2018). Explainable AI addresses this by quantifying feature contributions, SHAP deriving from game theory each feature's marginal contribution across all possible combinations (Lundberg and Lee, 2017). In heritage research, Dai et al. (2024) map affective responses to historic parks through sentiment computing, and Zhou et al. (2025) combine explainable methods with fine-tuned language models. Our approach extends this work by operationalizing assemblage theory empirically, measuring how relational configurations contribute to the model's predictions of expressed sentiment—a computational trace of emergent heritage value rather than a measure of causal efficacy in the site itself.

## 3 Materials and methods

This study employs a mixed-methods case study design, combining computational text analysis of social media data with qualitative contextual understanding of Xiaozhou Village's assemblage dynamics. Because the constructs at issue are psychological, we state at the outset what each computational step stands in for. The sentiment score indicates evaluative valence—how favorably a visitor appraises the setting—not emotion as experienced. The 18 lexicon features index what the visitor's account foregrounds, our observable proxy for the allocation of attention that Processing Fluency Theory and Attention Restoration Theory both concern. The engagement-depth code operationalises involvement with the place in the sense recreation specialization research uses the term, and author position the structural relation to the site that place attachment research treats as the source of place dependence. Each is a proxy measured from text rather than from the person, and the limitations of that choice are set out in §5.6.

### 3.1 Research design and case selection

We adopt an instrumental case study approach (Stake, 1995), in which Xiaozhou Village serves not as an intrinsically interesting object but as a strategic site for theoretical elaboration; instrumental designs select cases offering maximal analytical leverage for refining conceptual frameworks (Eisenhardt, 1989). Xiaozhou allows assemblage-topological theory to be tested and extended because it exemplifies tensions central to living heritage globally: rapid urbanization vs. cultural continuity, material degradation vs. affective vitality, and informal governance vs. heritage regulation.

Xiaozhou exemplifies what Flyvbjerg terms a “critical case”—one having strategic importance for theory development (Flyvbjerg, 2006)—in that it exhibits a pronounced material-affective disjuncture. We are careful about the evidence on the material side. Published reporting on Xiaozhou's waterways is largely an account of remediation rather than of decline—one widely circulated piece describes polluted orchards being restored into what it calls a lung for the city (Zhu, 2018)—and we have no independent monitoring series for the present period. What we can document is the degradation that visitors themselves name: dilapidation and litter appear in 110 posts and carry the second-largest negative attribution in the model (−0.583), and expressions of disappointment carry the largest (−0.766). The disjuncture examined here is therefore between what visitors report noticing and how they evaluate the place overall, not between measured environmental quality and reported satisfaction. Evaluations remain predominantly favorable—on Ctrip, the one platform in our corpus that records numerical ratings, the mean is 4.18 out of 5.0 (*n* = 138)—while cultural symbolism intensifies and tourism growth accelerates. This extremity on both dimensions provides optimal conditions for observing topological folding mechanisms. Figure 2 summarizes the research design. Figure 3 locates the village, its canal network and the spatial elements analyzed here.

**Figure 2:**
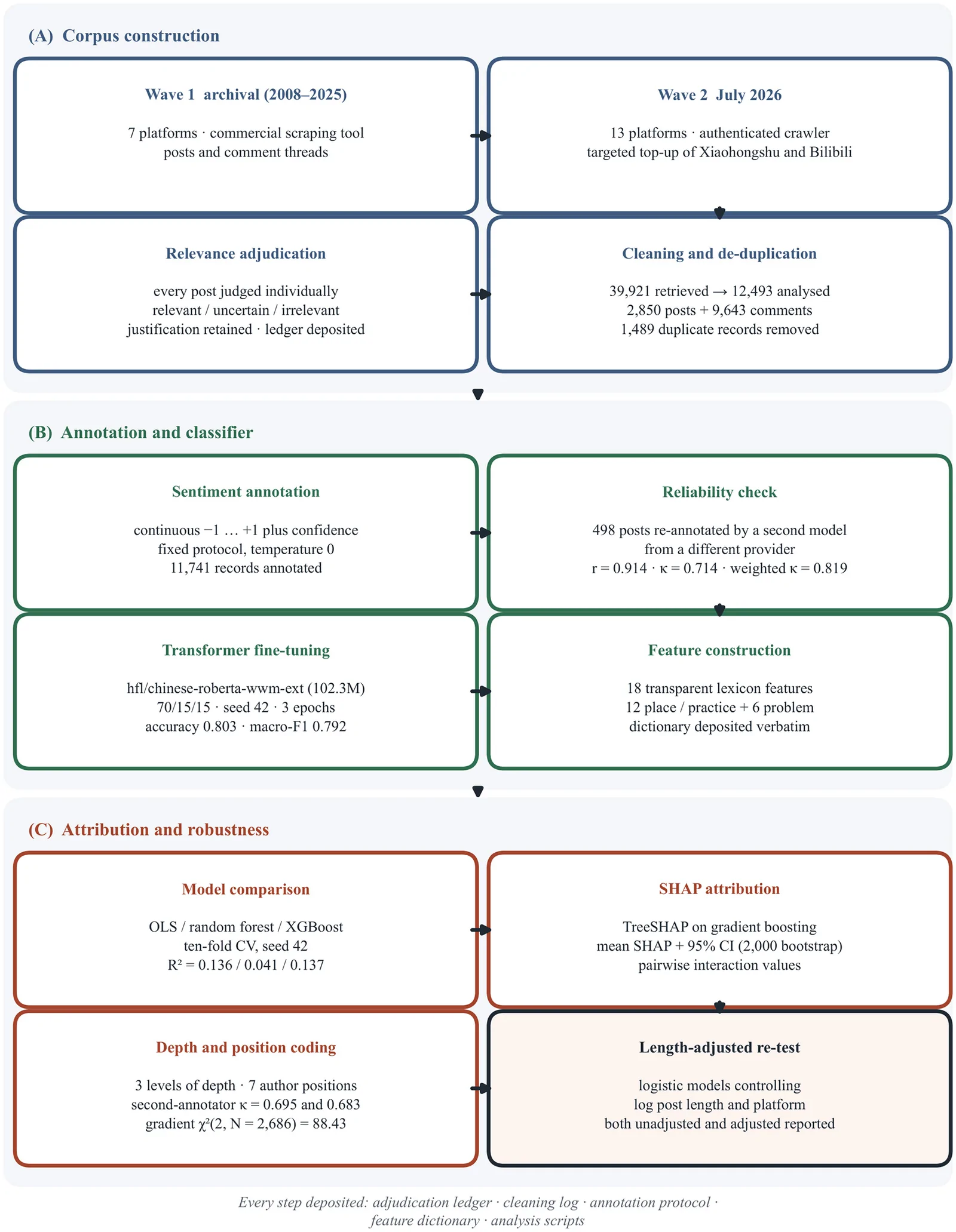
Mixed-methods research design. The three stages run from top to bottom. **(A)** covers corpus construction: two-wave sampling across thirteen platforms, per-record relevance adjudication, and cleaning and de-duplication reducing 39,921 retrieved records to 2,850 posts and 9,643 comments. **(B)** covers annotation and the classifier: sentiment annotation of 11,741 records under a fixed protocol, the cross-provider reliability check on 498 posts, transformer fine-tuning, and construction of the eighteen lexicon features. **(C)** covers attribution and robustness: model comparison, SHAP attribution, the engagement-depth and author-position coding with its own cross-provider reliability check, and the length-adjusted re-test.

**Figure 3:**
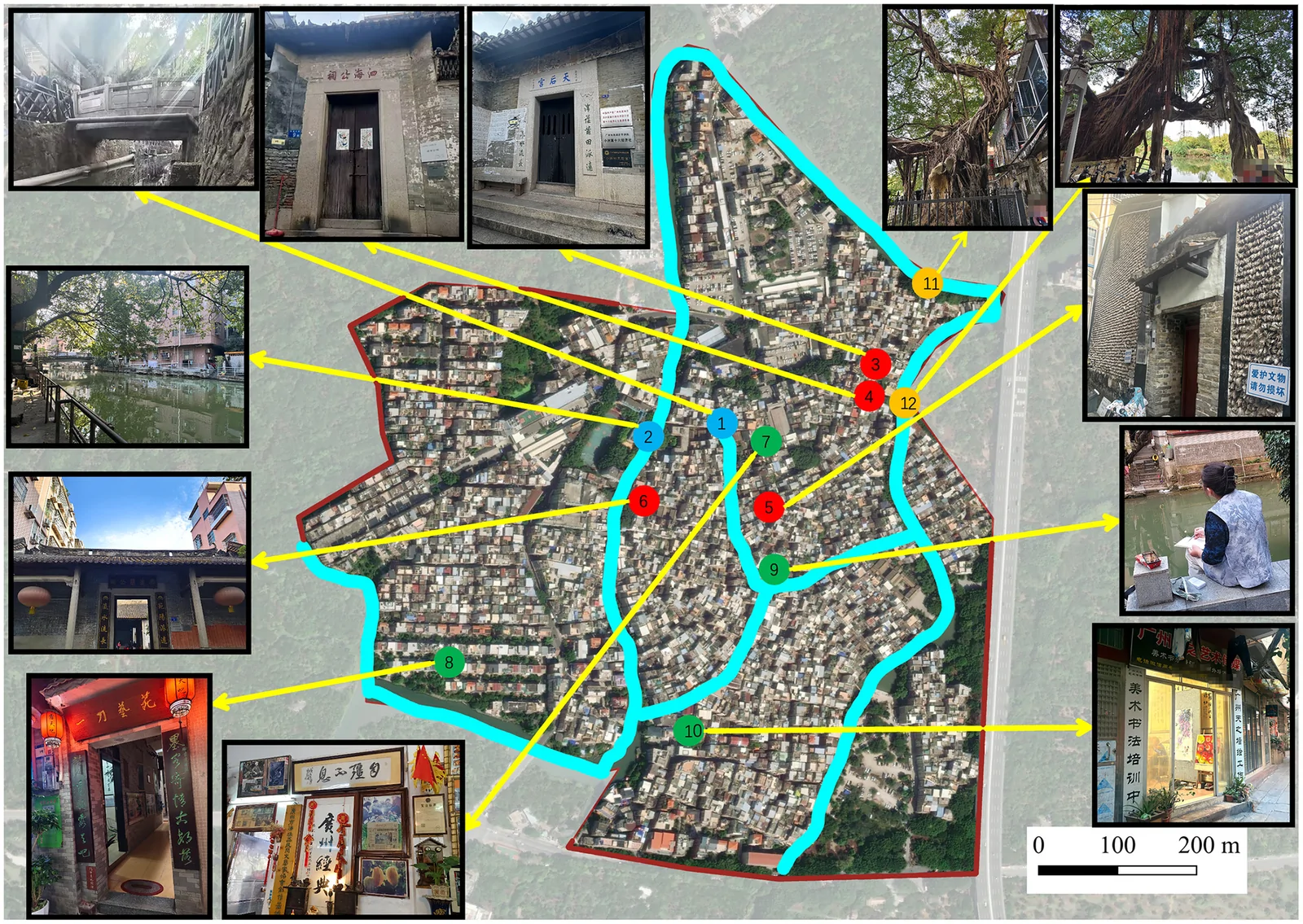
Xiaozhou Village: spatial elements and field photographs. The base is a satellite image of the village and its immediate surroundings; the dark red line marks the village boundary and the cyan overlay traces the canal network that gives the settlement its Lingnan water-town form. Twelve numbered markers locate the spatial elements on which the analysis draws, color-coded by category: the waterways and their stone bridges (blue, 1-2), heritage architecture including ancestral halls and oyster-shell masonry (red, 3-6), the artists' quarter with its studios, galleries and sketching sites (green, 7-10), and the old banyans (orange, 11-12). Each marker is linked by a leader line to a field photograph taken by the authors in December 2025. Faces and commercial contact details appearing in the photographs have been masked; nothing else in the images has been altered. Scale bar 200m. This figure reproduces an Esri World Imagery satellite basemap as a static image with the authors' overlays. Citation: Esri, World Imagery [basemap]. Source: https://server.arcgisonline.com/ArcGIS/rest/services/World_Imagery/MapServer. Licensed under Esri's terms for static maps in academic publications, which permit this use with attribution: https://doc.arcgis.com/en/arcgis-online/reference/static-maps.htm. Satellite basemap: Esri World Imagery. ©2026 Esri and its licensors. All rights reserved.

### 3.2 Multi-platform data collection

The corpus was assembled from publicly visible user-generated text mentioning “小洲村” (Xiaozhou Village) across 13 Chinese platforms, in two waves; Mafengwo's review and travelogue sections were retrieved separately, which is why 14 platform labels appear in the deposited corpus. The first retrieved archival material published between 2008 and 2025 from seven platforms using a commercial visual scraping tool; the second, in July 2026, used an open-source crawler framework for the seven platforms requiring an authenticated session and direct retrieval for the remainder, correcting two deficiencies in the first wave—thin coverage of Xiaohongshu, where the archival export had captured titles without post bodies, and of Bilibili. Comment threads were collected wherever the platform exposed them. One platform's comment interface (NetEase) was closed to retrieval throughout; this is reported as zero rather than imputed. No language filter was applied: retrieval was on the Chinese place name, and the corpus is Chinese-language throughout apart from two post-level records containing no Chinese characters at all, which were retained. All annotation, feature matching and modeling operate on the original Chinese; nothing was translated.

All records are publicly published user-generated content. Author names, profile links and IP-location fields were removed at ingestion and are absent from the deposited corpus; contact details appearing inside the published text itself—telephone numbers, e-mail addresses, messaging-service identifiers, third-party account handles and post URLs—were masked with fixed-length placeholders before deposit. The Ethics Statement sets this out in full. The ethical basis of the study and the terms under which the material was collected are set out in the Ethics Statement.

Keyword retrieval on a place name of this kind returns a large volume of irrelevant material: several other settlements in China share the name, and the string also matches a television drama, a personal name and general Guangzhou tourism content. Retrieval alone is therefore not a sampling frame. Every retrieved post was adjudicated individually for whether it concerns Xiaozhou Village in Guangzhou, by a large language model (deepseek-v4-flash, temperature 0) working from a written protocol that required a short justification for each decision; comments inherited the adjudication of their parent post. Each record was labeled relevant, uncertain or irrelevant, and the justification was retained. Measured irrelevance rates varied sharply by platform, from a few per cent on the Online Travel Agency (OTA) review platforms to the great majority of retrieved items on the news portal and one short-video platform—itself a methodological finding: keyword collection on these platforms is not usable without per-record adjudication. The full adjudication ledger is deposited, so that any individual decision can be inspected or overturned.

Records were removed if adjudicated irrelevant, if they carried no text, if they duplicated another record's content exactly—a substantial problem in the archival wave, where repeated crawls of the same task had produced 1,489 duplicated records—or if they were link spam or promotional boilerplate. Very short but genuine interactions were retained and flagged rather than deleted, so that analyses can include or exclude them. The pipeline reduced 39,921 retrieved records to an analytical corpus of 2,850 posts and 9,643 comments (12,493 records), of which 2,565 posts were adjudicated as clearly concerning Xiaozhou Village and 285 as uncertain; analyses were repeated with the uncertain records excluded without material change to the findings. The cleaning log records the number of records removed by each rule, and the cleaning script is deposited and re-runnable.

All material analyzed was published publicly by its authors. The study involved no recruitment of human participants, no private or restricted material and no interaction with the authors of the texts; under the applicable national and institutional guidance for research on public data it therefore did not require review by an institutional ethics board. Author names, profile links and IP-location fields were removed at ingestion, and contact details appearing inside the published text itself were masked before deposit.

### 3.3 Data processing and spatial element coding

We developed a hierarchical coding scheme combining theory-driven and data-driven categories, initially following Strauss and Corbin's grounded theory principles (Strauss and Corbin, 1990; Jia et al., 2024), and then operationalised it as an explicit string-matching dictionary so that every feature assignment is auditable against the source text. Material elements covered vernacular architecture (oyster-shell walls, ancestral halls, alleys), religious structures and modern interventions; ecological features covered water systems, vegetation and fauna.

Artistic practices covered creative activities (sketching, painting, photography, exhibitions), art venues and community references; affective-atmospheric descriptors covered positive affects (literary, quiet, relaxed, nostalgic, slow-paced), discovery narratives and temporal imaginaries; negative elements covered environmental issues (dirty, messy, polluted, crowded), commercialization concerns and infrastructure problems.

Eighteen features were retained for analysis: 12 concerning place elements and practices (sketching practice; art infrastructure and identities; photography; oyster-shell walls; ancient banyans; artistic atmosphere; traditional lanes and vernacular architecture; nostalgic affect; quiet and slow pace; hidden-gem discovery narrative; waterside ecology; food and consumption) and six concerning problems (commercialization; crowding; dilapidation and litter; access and parking; cost and ticketing; disappointment). Each is a binary indicator produced by the dictionary, whose full contents are reproduced in the supplementary material and deposited as a machine-readable file, so that feature construction can be reproduced exactly rather than described in prose. Using a transparent lexicon rather than a learned representation is deliberate: it keeps the attribution analysis interpretable at the cost of some predictive power, and it makes every assignment checkable. Platform indicators and log post length were included as covariates; the latter matters because posts differ markedly in length and longer texts are mechanically more likely to trigger any lexicon feature.

Three of these features concern art and are deliberately kept apart, because an earlier version of the dictionary did not keep them apart and the difference proved consequential. Sketching practice captures records of someone drawing or painting; art ecology captures the village's artistic infrastructure and the identities attached to it—studios, the nearby academy, examination ateliers, artists and art students; artistic atmosphere captures ambient description. Xiaozhou adjoins a major art academy, so the institutional vocabulary is dense in this corpus and appears most often when studios are closing rather than when anyone is at work. A combined feature therefore measures the discussion of the art economy at least as much as it measures the practice, and §4.3 reports what happens to the results when the two are separated. We report the separated specification throughout.

Two further variables were coded from the text of each post; because the first carries the main argument, its rule is given here in full. Depth of engagement was coded on three levels: level 3 where the author reports repeated visits or extended dwelling—having lived in the village, studied painting there, rented a studio, visited many times, or writing as someone who came from Xiaozhou; level 2 where the author reports a single purposive visit with substantive description of what was seen or done; level 1 where the record is a passing mention, a repost, or pure information with no evidence of the author's own visit. Posts for which none of these could be established were coded 0 and excluded from the depth analyses (164 of 2,850). Author position was coded into seven categories—general visitor, art student, artist, resident, merchant, media or promotional account, and undetermined. Both were coded by the same model and protocol used for sentiment, each decision accompanied by a short justification that is retained in the deposit; the full instruction is reproduced in the supplementary material. Because the depth code carries the main argument, it was subjected to the same cross-provider reliability check as the sentiment annotation: a second, independently developed model from a different provider (doubao-seed-2-1-turbo) re-coded a sample of 499 posts stratified by depth level, under the identical protocol at temperature 0. Agreement was substantial and comparable to that obtained for sentiment: for depth, 79.0% exact agreement, Cohen's κ = 0.695 and quadratic-weighted κ = 0.724; for author position, 76.4% exact agreement and κ = 0.683.

### 3.4 BERT-based sentiment modeling

Sentiment annotation and modeling rest on Chinese-BERT-wwm (Whole Word Masking), a pre-trained language model fine-tuned for sentiment classification (Cui et al., 2020). Bidirectional context modeling—each word's representation incorporates both preceding and following text—captures evaluative language that lexicon matching and bag-of-words models miss (Devlin et al., 2019; Dai et al., 2024): “真是'干净'啊”/really “clean” [sarcastic] is read as negative despite the positive keyword.

Sentiment was first annotated at the record level on a continuous scale from −1 (strongly negative) through 0 (neutral or purely informational) to +1 (strongly positive), together with a confidence value and a short justification, by a large language model (deepseek-v4-flash) under a fixed protocol at temperature 0; the protocol text is reproduced in the supplementary material. Annotation reliability was assessed by having a second, independently developed model from a different provider (doubao-seed-2-1-turbo) re-annotate a stratified sample of 498 posts: agreement was high (Pearson *r* = 0.914; three-class agreement 80.9%; Cohen's κ = 0.714; quadratic-weighted κ = 0.819).

To obtain a sentiment model that can be redeployed and inspected independently of any annotation service, we fine-tuned a Chinese pre-trained transformer (hfl/chinese-roberta-wwm-ext, 102.3M parameters) as a three-class classifier (negative ≤ −0.2, neutral, positive ≥ +0.2; the cut-point is conventional rather than derived, and §4.3 reports that the substantive results are unchanged across cut-points from ±0.1 to ±0.4). Of the 12,493 records in the analytical corpus, 11,741 carried text usable for annotation and entered the modeling—all 2,850 posts and 8,891 of the comments; the remaining 752 comments were emoji-only, sticker-only or otherwise empty of scoreable text once boilerplate had been stripped, and are retained in the deposited corpus but excluded from every model. These 11,741 records were split 70/15/15 into training (*n* = 8,218), validation (*n* = 1,761) and test (*n* = 1,762) partitions by stratified sampling with random seed 42. Training ran for three epochs at a learning rate of 2 × 10^−5^, batch size 32, maximum sequence length 160, with linear warmup over the first 10% of steps, on Apple silicon using the Metal Performance Shaders backend; results on other hardware may differ in the last decimal place. Test-set performance was accuracy 0.803, macro-precision 0.793, macro-recall 0.792, macro-F1 0.792 and weighted-F1 0.802, with per-class F1 of 0.78 (negative), 0.75 (neutral) and 0.85 (positive). The fine-tuned classifier was then applied to the whole corpus; its continuous score, defined as P(positive)-P(negative), is the dependent variable in the attribution analysis and correlates with the annotation scores at *r* = 0.864.

Annotation and feature construction are deliberately independent: sentiment is annotated from the text, while the features of §3.3 come from the explicit dictionary, so the model is not asked to explain its own judgments.

### 3.5 SHAP attribution analysis

For each post we constructed a feature vector recording the presence or absence of each of the 18 dictionary features, together with platform indicators and log post length. Binary rather than frequency encoding was used: the quantity of interest is whether an element enters the account at all, not how often it is named.

Three regression models were compared under ten-fold cross-validation with random seed 42: ordinary least squares, a random forest (500 trees) and gradient boosting (XGBoost; 600 rounds, maximum depth 4, learning rate 0.05, subsample 0.8, column subsample 0.8). Cross-validated *R*^2^ was 0.136 for linear regression, 0.041 for the random forest and 0.137 for gradient boosting; with the annotation score as the dependent variable the corresponding values were 0.177, 0.099 and 0.172. Gradient boosting was selected for attribution. An *R*^2^ of this magnitude is the share of variance in sentiment that 18 explicit textual cues can account for: it identifies which cues the model weighs, not the determinants of visitor experience as a whole.

We employed TreeSHAP, which computes exact Shapley values for tree-based models (Lundberg et al., 2020). For a given post and feature, the SHAP value is that feature's contribution to the model's prediction for that post, defined so that the prediction equals the model's average prediction over the corpus plus the sum of the SHAP values of all features present.

SHAP analysis yields global feature importance (mean absolute SHAP values), directional contributions (mean SHAP values, showing whether a feature pushes sentiment positive or negative), interaction effects (how one feature's impact varies with another's presence), and instance-level explanations of why particular posts scored as they did. All group contrasts reported below were also re-estimated in logistic models controlling for log post length and platform; both unadjusted and adjusted results are reported.

## 4 Results

We state first what the analysis is meant to test and what would count against it. The general form of the topological folding hypothesis predicts that engagement in embodied practice is associated with environmental deficits receding from the account, whoever is doing the practicing; the conditional form predicts that the association depends on the depth of the practice. The two make opposite predictions about the share of negative expression among the most deeply engaged authors: the general form is supported if that share is flat or falling across levels of engagement depth, the conditional form if it rises. If the rise disappeared once length was controlled, neither form would be supported: the result would be an artifact of expression volume rather than of attention.

The sections that follow are organized by research question: §4.1 reports the composition of the corpus and model performance and §4.2 the feature attributions, which together answer RQ1; §4.3 sets activity type against depth of engagement to address RQ3, including what happened when one of those measures was found to be mis-specified; §4.4 turns to who is speaking and §4.5 draws the findings together. RQ2 is answered across §4.3 and §4.4 jointly, and taken up again in the Discussion.

### 4.1 Descriptive statistics and platform variations

The analytical corpus comprises 2,850 posts and 9,643 comments (12,493 records) drawn from 13 platforms. Weibo (793 posts), Xiaohongshu (505), Zhihu (369), the OTA review platforms (Qunar 307, Ctrip 138, Mafengwo 90), Tieba (251) and Bilibili (197) account for the bulk of the post-level corpus, with the remainder distributed across Douyin, Toutiao, WeChat public accounts, Kuaishou and NetEase. Of the post-level records, 2,565 were adjudicated as clearly concerning Xiaozhou Village and 285 as uncertain; both are retained, and analyses were repeated with the uncertain records excluded without material change to the findings.

Sentiment annotation yielded a distribution of 62.1% positive, 23.1% neutral and 14.8% negative at the post level. Annotation reliability was assessed by having a second, independently developed large language model from a different provider re-annotate a stratified sample of 498 posts. Agreement between the two annotators was high: Pearson *r* = 0.914, three-class agreement 80.9%, Cohen's κ = 0.714, and quadratic-weighted κ = 0.819.

The fine-tuned Chinese transformer classifier achieved test-set accuracy of 0.803 and macro-F1 of 0.792 (per-class F1: negative 0.78, neutral 0.75, positive 0.85) on 1,762 held-out records; its continuous sentiment score correlates with the annotation scores at *r* = 0.864. Ten-fold cross-validation of the attribution models gave *R*^2^ of 0.136 for linear regression, 0.041 for random forest and 0.137 for gradient boosting (annotation score as dependent variable: 0.177, 0.099 and 0.172; Table 1). Gradient boosting was selected for SHAP attribution. An *R*^2^ of this magnitude means that the 18 lexicon features account for a modest share of the variance in sentiment: they identify which explicit textual cues the model weighs, not the determinants of visitor experience as a whole.

**Table 1:** Machine learning model performance comparison (ten-fold cross-validation, post level, *n* = 2,850).

Model	*R* ^2^	RMSE	MAE
Linear regression	0.136	0.636	0.521
Random forest	0.041	0.670	0.497
Gradient boosting (XGBoost)	0.137	0.635	0.495

### 4.2 SHAP feature contributions and topological dynamics

Table 2 reports each feature's mean SHAP value, its average marginal contribution to predicted sentiment across posts in which the feature is present, with 95% confidence intervals (CI) from 2,000 bootstrap resamples.

**Table 2:** SHAP feature attributions to predicted sentiment (post level, *n* = 2,850).

Rank	Feature	*n* posts	Mean SHAP	95% CI	Mean SHAP (annotation score)
1	Hidden-gem discovery narrative	185	+0.221	[+0.210, +0.232]	+0.189
2	Food and consumption	376	+0.205	[+0.198, +0.211]	+0.155
3	Quiet, slow pace	289	+0.157	[+0.147, +0.167]	+0.152
4	Ancient banyan trees	101	+0.152	[+0.137, +0.166]	+0.105
5	Oyster-shell walls	220	+0.150	[+0.144, +0.156]	+0.080
6	Photography	295	+0.097	[+0.091, +0.102]	+0.087
7	Sketching practice	98	+0.087	[+0.075, +0.100]	+0.051
8	Nostalgic affect	147	+0.063	[+0.050, +0.075]	+0.025
9	Literary/artistic atmosphere	473	+0.055	[+0.049, +0.062]	+0.047
10	Waterside ecology	691	+0.050	[+0.047, +0.053]	+0.043
11	Traditional alleys	402	+0.002	[−0.002, +0.006]	+0.016
12	Art infrastructure and identities	454	−0.007	[−0.013, −0.001]	+0.001
13	Over-commercialization	68	−0.042	[−0.075, −0.012]	−0.042
14	Access and parking	139	−0.088	[−0.103, −0.073]	−0.041
15	Crowding	46	−0.100	[−0.126, −0.077]	−0.062
16	Cost and ticketing	123	−0.111	[−0.126, −0.095]	−0.150
17	Dilapidation and litter	110	−0.583	[−0.618, −0.550]	−0.436
18	Disappointment	86	−0.766	[−0.810, −0.721]	−0.540

Mean SHAP values represent the average marginal contribution of each feature to the model's predicted sentiment across all posts in which the feature is present; 95% confidence intervals are from 2,000 bootstrap resamples. The final column reports the same quantity with the annotation score as the dependent variable, as a robustness check. Held-out test performance of the selected model: *R*^2^ = 0.160, RMSE = 0.637, MAE = 0.494 (*n* = 570). SHAP values quantify contributions to model predictions and do not establish causal effects.

These attributions run against the study's initial expectation, though not in the direction we first read them. The strongest positive attributions attach not to any artistic activity but to narratives of discovery [+0.221, CI (0.210, 0.232)], everyday consumption [+0.205, CI (0.198, 0.211)] and quietness and slow pace [+0.157, CI (0.147, 0.167)]. Material heritage elements occupy intermediate positions: ancient banyans +0.152, oyster-shell walls +0.150, waterside ecology +0.050. The two embodied practices both sit on the positive side—photography +0.097 [CI (0.091, 0.102)] and sketching practice +0.087 [CI (0.075, 0.100)]—while the feature capturing the village's artistic infrastructure and the identities attached to it (studios, academies, examination preparation, artists) carries a small negative attribution [−0.007, CI (−0.013, −0.001)]. The strongest negative attributions attach to expressions of disappointment (−0.766) and to physical dilapidation and litter (−0.583), both far exceeding in magnitude any positive feature.

One decision behind these figures deserves to be stated, because an earlier version of this analysis reached the opposite conclusion without it. Our first lexicon placed studio, academy, art-examination and graffiti in the same feature as sketching, painting and drawing. Xiaozhou adjoins a major art academy and is full of rented studios and examination ateliers, and those four terms turn out to appear most often when the studios are closing rather than when anyone is painting. Under the combined feature, sketching appeared to carry a negative attribution and to be associated with more negative expression; separating the two—records of someone painting on one side, the village's art infrastructure and identities on the other—removes that result entirely (the separation and its consequences are reported in §4.3). We report the corrected specification throughout and treat the episode as instructive: in lexicon-based work on user-generated text, a feature that mixes an activity with the institutions surrounding it will measure whichever of the two is more often written about.

The strongest pairwise interactions in the corpus are of modest magnitude and do not follow the art-amplifies-nature pattern anticipated at the outset: the largest mean absolute interactions involve artistic atmosphere with dilapidation and litter, waterside ecology with access and parking, and a quiet pace with everyday consumption. Elements therefore do derive part of their weight from relational configuration rather than intrinsic properties—assemblage theory's core proposition survives—but the couplings differ from those hypothesized.

Read together, the attributions do not support a general threshold argument in which positive drivers outweigh negative ones by a fixed ratio: the two strongest attributions in the corpus are negative, not positive, and exceed the strongest positive attribution by roughly a factor of four. What sustains the predominantly positive sentiment is therefore not the magnitude of positive drivers but the rarity with which the strong negative features are invoked at all: disappointment appears in 3.0% of posts and dilapidation in 3.9%, so the question becomes who invokes them.

The predicted interaction—artistic engagement attenuating the negative weight of dilapidation—is not present in the data, and neither is its reverse. With post length and platform controlled, sketching practice shows no association with mentions of dilapidation (OR = 1.12, *p* = 0.795), nor does photography (OR = 1.05, *p* = 0.863); post length is by far the strongest predictor of whether any lexicon feature is triggered at all (OR = 2.01, *p* < 0.00001). What survives adjustment is a difference between the two practices in overall negative expression, running in one direction only: photography is associated with markedly less of it (OR = 0.43, p = 0.0006), while sketching practice shows no detectable association (OR = 0.75, *p* = 0.381). The buffering hypothesis is therefore not so much reversed as displaced: what organizes attention to environmental decline in this corpus is not which artistic activity a visitor reports.

### 4.3 Depth of engagement

The organizing variable is how deeply the visit is invested rather than what is done during it. Each post was coded for the relationship to the site it records: passing mention or pure information, a single purposive visit, or repeated visits or extended dwelling (*n* = 1,797, 679 and 210). The share of negative posts rises across the three levels. On the annotation score, which is what Table 3 reports, the shares are 10.7, 22.8 and 28.6% (χ^2^(2, *N* = 2,686) = 88.43, *p* < 0.00001; Spearman ρ = +0.181, *p* < 0.00001); on the classifier score they are 11.1, 22.8 and 27.1% (χ^2^(2, *N* = 2,686) = 76.37, *p* < 0.00001; Spearman ρ = +0.169). The models below use the classifier score. Unlike the activity features, this gradient is not an artifact of how much each group writes: median post length across the three levels is 87, 112 and 119 characters, a far narrower spread than between activity groups, and controlling for length and platform gives odds ratios of 2.12 and 3.25 (both *p* < 0.00001), length itself carrying none (OR = 0.97, *p* = 0.523). Adding the activity features leaves the gradient essentially unchanged (OR = 2.21 and 3.29, both *p* < 0.00001; length OR = 1.00, *p* = 0.976), photography retaining its independent negative association and sketching showing none. Depth and activity type are therefore separable, and it is depth that carries the pattern. On the annotation score the same models give 2.17 and 3.58, and 2.26 and 3.66 with the activity features entered—slightly larger, so the outcome measure does not carry the result; and with the sentiment cut-point moved from ±0.1 to ±0.4 the adjusted odds ratio at the deepest level stays between 3.20 and 3.29 (*p* < 10^−10^ throughout).

**Table 3:** Sentiment and environmental criticism by depth of engagement and author position.

Group	*n*	Mean sentiment	% negative	% mentioning dilapidation
Passing mention/information only	1,797	0.292	10.7%	2.1%
One purposive visit	679	0.295	22.8%	8.8%
Repeated visits or dwelling	210	0.196	28.6%	2.9%
Author position				
Local residents	72	0.048	33.3%	6.9%
General visitors	782	0.274	22.8%	7.9%
Art students	62	0.250	21.0%	0.0%
Undetermined	891	0.234	15.3%	2.0%
Artists	46	0.301	13.0%	2.2%
Media/promotional accounts	641	0.385	7.5%	2.7%
Commercial accounts	192	0.333	1.0%	0.0%

Depth of engagement and author position were coded from the text of each post; depth could be determined for 2,686 posts and author position for 1,795 of them (66.8%). Mean sentiment and the negative share are computed on the annotation score; the adjusted models in §4.3 and §4.4 use the classifier score, and both specifications are reported there. The share of negative posts rises monotonically with depth (χ^2^(2, *N* = 2,686) = 88.43, *p* < 0.00001; Spearman ρ = +0.181). Because posts differ in length and longer texts are mechanically more likely to trigger any dictionary feature, every group contrast is re-estimated controlling for log post length and platform; see §4.3 and §4.4.

One feature of the coding scheme requires disclosure, because it could have produced the gradient by construction. The instruction for the deepest level listed studying painting in the village among its markers, alongside having lived there, renting a studio, visiting repeatedly, and writing as someone who came from Xiaozhou; since art-related content also enters the feature set, the two are not fully independent by design. We therefore re-estimated the gradient on the subset of posts containing neither a painting-practice term nor an art-institution term, which removes 483 of the 2,686 coded posts. The gradient is undiminished: negative shares of 11.0, 23.7 and 27.5% across the three levels (χ^2^(2, *N* = 2,203) = 68.81, *p* < 0.00001; Spearman ρ = +0.177), and with post length and platform controlled, odds ratios of 2.26 and 3.50 at the second and third levels (both *p* < 0.00001), slightly larger than in the full sample. Whatever the depth coding captures, it is not an artifact of the art vocabulary that appears in one of its markers.

One quantity related to the gradient does not rise monotonically, and we report it as it stands: the rate at which any environmental problem is named peaks at the middle level rather than the highest (11.1, 24.3, 16.2%), and dilapidation, the most consequential problem category, is named in 2.1, 8.8 and 2.9% of posts across the three levels. Reading the posts suggests why. Authors describing a single dedicated visit write itemized accounts naming specific conditions—the water, the litter, the queue—whereas the most engaged authors more often write in general terms about change, that the village is not what it was, which a dictionary built to capture named conditions does not register. The share of negative expression does not depend on any condition being named and carries the gradient cleanly, and the analyses below use it rather than the problem-naming rate.

Because the separation of the sketching feature described in §3.3 changed the direction of a reported result, we set out its consequences in full. Under the combined lexicon, 228 posts were coded as sketching-related. Of these, 98 contain a word denoting the act of painting or drawing; the remaining 130 entered only through studio, academy, art-examination or graffiti. Posts recording the act carry a negative share of 13.3%, indistinguishable from the 14.6% among posts containing neither kind of term (χ^2^(1, *N* = 2,720) = 0.05, *p* = 0.815). Posts entering only through the institutional terms carry 26.2%, significantly higher than that baseline (χ^2^(1, *N* = 2,752) = 11.86, *p* = 0.0006), and the two subsets differ from each other (Fisher's exact *p* = 0.020, OR = 0.43). The pattern replicates under the annotation-score specification. In the model that also controls engagement depth and the other activity features, neither subset retains an association with negative expression (OR = 0.75, *p* = 0.381 and OR = 1.21, *p* = 0.235); with only post length and platform controlled the painting-act subset likewise shows none (OR = 0.92, *p* = 0.782). Either way the corrected analysis reports no activity effect on that side. We record this because the combined feature had produced an apparently robust finding in the opposite direction, and because the failure mode is general: where an activity and the institutions that host it share a vocabulary, the feature measures whichever is more often discussed.

Promotional accounts cluster at the shallow end—600 media and 171 merchant posts fall in the first depth level, where their negative shares are 8.0 and 1.6%—so the gradient was re-estimated with author position as a further control. The contrast that carries the argument is unchanged: with length, platform, activity type and author position controlled, the odds of negative expression among those who return repeatedly or stay are 2.14 times those of passers-by (*p* = 0.0005); excluding media and merchant accounts gives 2.54 (*p* < 0.00001); and photography remains associated with markedly less negative expression (OR = 0.42, *p* = 0.0004). The intermediate level is the part that depends on who is writing: it falls from 2.21 to 1.49 (*p* = 0.018) once position is controlled, and among the 782 posts by ordinary visitors alone it is not separable from unity (OR = 1.31, *p* = 0.223; the deepest contrast there is OR = 1.93, *p* = 0.079, on 180 negative cases). We therefore state the result as a contrast between sustained involvement and passing contact rather than as a strictly monotonic gradient. This is the lesson of the lexicon separation above, applied on the outcome side: where a category and the population that uses it are correlated, an estimate that omits the population recovers part of the population. All four specifications are deposited.

The checks that failed to catch the problem were the ones that usually suffice: confounding (post length, platform), a second outcome measure, an independently collected comment corpus, and co-occurrence with related features. The result survived all four, because all four ask whether an association is robust and none asks whether the variable measures what its name says.

### 4.4 Author position

Coding also distinguished the position from which each author writes. Among posts where this could be determined, locally resident authors are the most critical group in the corpus (*n* = 72; mean sentiment 0.048; 33.3% negative), followed by general visitors (*n* = 782; 22.8%) and art students (*n* = 62; 21.0%); at the opposite end, commercial accounts (*n* = 192) register 1.0% and media or promotional accounts (*n* = 641) 7.5%. One group runs against the pattern and we report it as such: authors identifiable as established artists (*n* = 46) record the third-highest mean sentiment of any group (0.301, behind media accounts at 0.385 and merchants at 0.333) and a negative share of 13.0%, below that of general visitors, and they mention environmental problems in only 6.5% of their posts, less often than any other group except merchants (4.7%). Two readings are available and the data do not separate them: established artists in Xiaozhou typically hold studio or retail premises, which places them closer to the commercial accounts than to the art students in what they can afford to say in public; alternatively, those for whom conditions had become unworkable may already have left, so that the group we observe is the one that stayed. The cell is small and we draw no conclusion from it, but it marks the clearest limit of the gradient: depth of investment predicts critical expression among those who write as practitioners, not among those who write as proprietors. Table 3 reports the three depth levels and the seven author positions together. These shares are unadjusted. Re-estimated on the classifier score with log post length and platform controlled and general visitors as the reference category, residents remain more likely to express negatively (OR = 1.77, *p* = 0.042), merchants and media accounts much less so (OR = 0.08 and 0.29, both *p* < 0.0001), while art students do not differ (OR = 1.25, *p* = 0.493) and the artists' lower rate does not survive adjustment (OR = 0.52, *p* = 0.182)—which is the more conservative reading of that exception. Author position could not be determined for 33.2% of the posts for which coding was attempted, and the figures above apply only to the remainder.

Two features of this pattern bear on corpus-based heritage research. Resident voices are not absent from platform discourse, and where they appear they are systematically more critical than visitor voices, so a corpus reporting only aggregate sentiment will understate the severity of locally perceived decline. A substantial part of the positive sentiment on these platforms is supplied by accounts with a commercial interest in the site, a concrete form of the performativity that makes social-media corpora non-equivalent to interview or survey data.

Figure 4 pairs the elements carrying the largest positive attributions—the Qing-era oyster-shell masonry, the old banyans, the canal network that gives the village its Lingnan water-town form—with the other register a few 100 m downstream: refuse and turbid water in the same canal system, cabling and laundry strung across the canal frontage, and a cafe now occupying premises that once housed workshops and studios. All six photographs were taken during a single field visit within roughly 420 by 460 meters; the two rows are not different places but the same village photographed within walking distance of itself. Over-commercialization is named often by long-dwelling practitioners, yet its attributed weight is the smallest of any negative feature: how often something is said and how much it moves predicted sentiment are different quantities.

**Figure 4:**
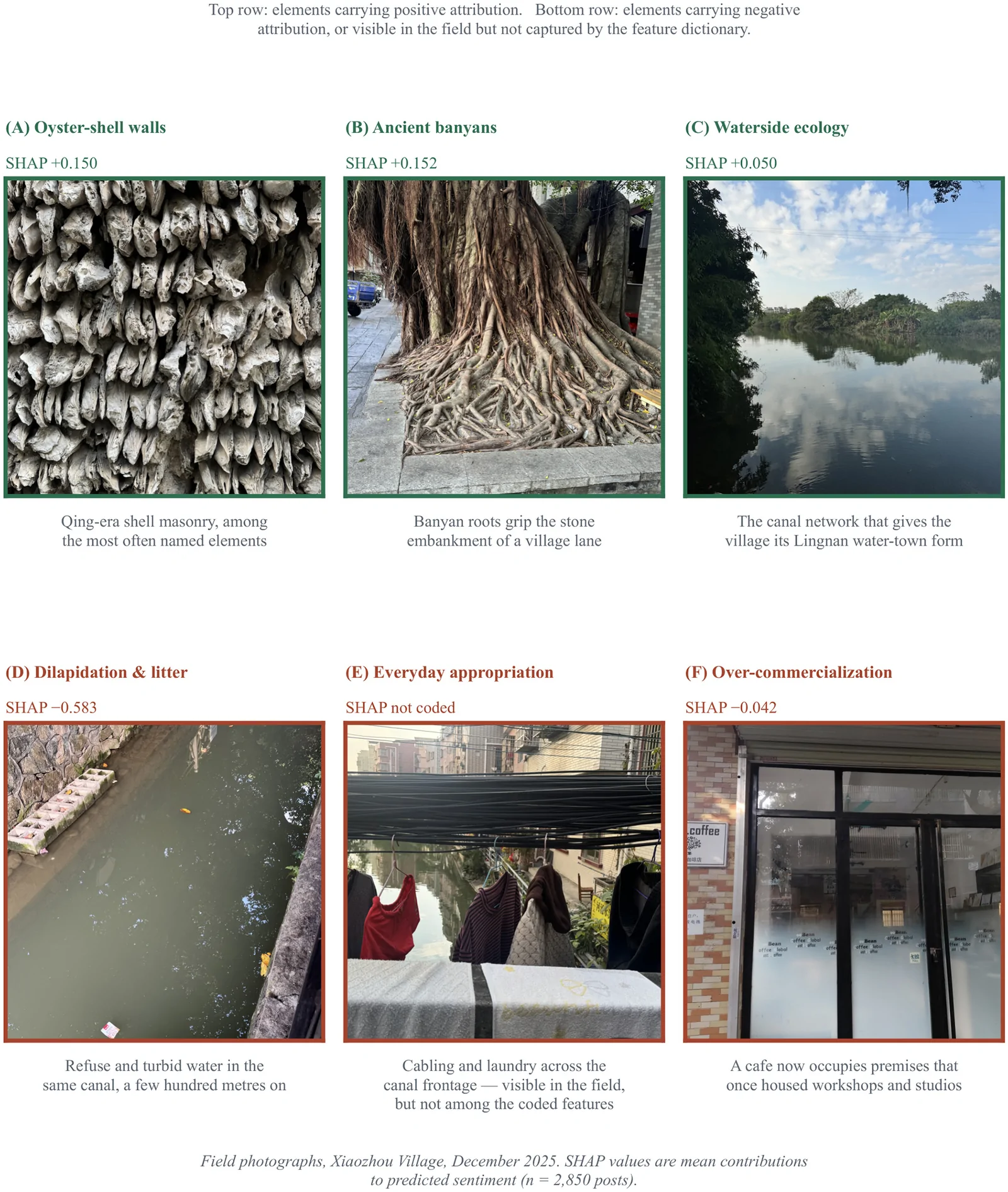
Two registers of the same village. **(A–C)** show elements carrying positive attribution in the model: Qing-era oyster-shell masonry (SHAP +0.150), the old banyans whose roots grip the stone embankments of the village lanes (+0.152) and the canal network that gives the village its Lingnan water-town form (+0.050). **(D–F)** show the other register: refuse and turbid water in the same canal system a few 100 m downstream, the feature carrying the second-largest negative attribution in the corpus (−0.583); cabling and laundry strung across the canal frontage, which is conspicuous in the field but is not among the 18 coded features; and a cafe now occupying premises that once housed workshops and studios (−0.042). All six photographs were taken by the authors during a single field visit in December 2025, within an area measuring roughly 420 by 460 m.

### 4.5 Synthesis of findings

Collectively, these results require the assemblage-topological framework to be stated conditionally. Multi-platform coverage confirms that sentiment about Xiaozhou remains predominantly positive despite widespread awareness of environmental degradation—the empirical puzzle that motivated the study stands. Attribution analysis, however, locates the sources of that positivity in narratives of discovery, everyday consumption and ambient quietness rather than in artistic practice, and shows the strongest negative attributions to exceed the strongest positive ones while appearing far less often. The contrast between photography and sketching, which holds at both the post and the comment level and under both dependent variables, together with the monotonic rise of negative expression across three levels of engagement depth, indicates that topological folding does not operate in a single direction: brief visual consumption folds degradation away, sustained creative investment folds it into view.

## 5 Discussion

### 5.1 Attentional reorganization as a depth-dependent cognitive-affective mechanism

Two features of the results—the strongest negative contributions (disappointment −0.766, dilapidation −0.583) exceeding the strongest positive ones while appearing far less often, and the share of negative expression rising monotonically with depth of engagement (10.7, 22.8, 28.6%; *p* < 0.00001)—reflect the process this paper calls topological folding: a reorganization of attention in which some elements achieve cognitive salience while others recede to background, in a direction that varies with how deeply the visit is invested. Three dynamics organize the account that follows: selective attention amplification (Processing Fluency Theory), activity-specific sensitization (Cognitive Dissonance Theory) and emergent atmospheric production (Attention Restoration Theory).

Selective attention amplification occurs when embodied artistic practices intensify awareness of specific material elements: for the visitor who sketches, oyster-shell walls become compositional subjects demanding observation rather than background texture. What the present findings add is that the amplification is not selective as to valence—the same sustained observation also registers the refuse at the wall's base. Attention trained on a place returns more of everything the place contains, which is why the practice most demanding of observation is also the practice whose accounts most often name what has gone wrong.

Depth-specific sensitization describes what the data show. The interaction the initial framework predicted, artistic engagement attenuating the weight of dilapidation, does not appear: with post length controlled, no activity type is associated with how often environmental problems are named (sketching practice OR = 1.12, *p* = 0.795; photography OR = 1.05, *p* = 0.863; length itself OR = 2.01, *p* < 0.00001). What organizes the pattern is how deeply the visit is invested: the odds of negative expression among those who return repeatedly or stay are 3.29 times those of passers-by with length, platform and activity type controlled, and 2.14 times with the author's position controlled as well (§4.3). Sustained involvement does recalibrate aesthetic criteria, but upward: weathered surfaces read as patina only while the surrounding conditions still permit the work to be done. When they no longer do—when, as one long-dwelling art student reports a painter of his acquaintance saying, “you simply cannot paint in Xiaozhou now—squeeze out the paint and within 10 minutes it is covered in dust,” and that “many painters have already left” (forum, 2013)—the same sensibility that once found patina registers damage. A visitor returning after seven or 8 years finds the studios and craft shops that once lined the lanes “all gone” (lifestyle-sharing platform, 2025). Photography runs the other way: briefer and more visually selective, it is associated with markedly less negative expression (OR = 0.43, *p* = 0.0006; 7.8% negative vs. 16.0% among other posts, *p* = 0.0003).

Effort justification, the better-known prediction of Cognitive Dissonance Theory, runs the other way: having invested effort in a choice, people revise their evaluation upward, so those who have given most to Xiaozhou should be the least willing to see what has gone wrong. The corpus does not behave that way. Effort justification appears to operate on the practice rather than the place: what the long-dwelling practitioner defends is the value of painting here, and that commitment makes the deteriorating conditions intolerable rather than invisible. Dissonance is resolved not by finding the village acceptable but by saying loudly that it has changed: the attachment survives as a grievance.

Emergent atmospheric production concerns qualities exceeding the sum of components. Artistic atmosphere retains an independent, if modest, positive attribution [+0.055, CI (0.049, 0.062)] once the elements it might be reducible to—galleries, ancestral halls, artistic practices—are accounted for, which suggests that assemblages generate atmospheres that then guide what visitors attend to next. That it is small relative to the attributions attaching to discovery narratives and to quietness is telling: what carries affective weight here is less the label “artistic village” than the qualities of encounter it promises.

### 5.2 Processing fluency and its boundaries in environmental engagement

Processing Fluency Theory illuminates engagement in heritage settings, and also shows where its reach ends. According to Reber et al. (2004), stimuli that are processed more easily tend to be evaluated more positively, and several pathways of the Xiaozhou assemblage make the village easy to read (Deleuze and Guattari, 1988). The account fits visually oriented practice closely: posts reporting photography carry significantly more positive sentiment than the rest (*t* = 7.50, *p* < 0.0001, Cohen's *d* = 0.38), photographic framing selecting composable views that the semiotic coding supplies ready-made. Sketching does not. Posts reporting sketching practice are not distinguishable from other posts (*t* = 1.65, *p* = 0.101), and the marginal contribution of sketching practice to the model's prediction is positive but modest [SHAP +0.087, 95% CI (0.075, 0.100)], close to that of photography (+0.097). Fluency therefore describes one end of a gradient of engagement, not a general property of embodied artistic practice.

The village is territorialized primarily through semiotic coding: media, guidebooks, influencer posts and official materials frame it as “艺术家村” (artists' village) and “岭南水乡” (Lingnan water settlement), setting expectations about elements and practices—oyster-shell walls (7.7% of posts) and a literary atmosphere (16.6%) recur across visit accounts despite being a minority of the physical fabric. Two deterritorializations cut across this coding: artistic flows turning vernacular architecture into galleries, and commercial flows replacing residences and workshops with cafés, boutiques and homestays.

The two deterritorializations are not registered in the same terms. The largest positive marginal contributions to predicted sentiment come from hidden-gem discovery narratives (+0.221), food consumption (+0.205) and a quiet, slow pace (+0.157), while over-commercialization contributes negatively [−0.042, CI (−0.075, −0.012)]: not all deterritorializations are equivalent losses of heritage value. Artistic flows resonate with the existing coding rather than contradicting it: sketching old buildings and photographing vernacular life perform heritage appreciation. What the present analysis adds is that performing it does not insulate the performer from the village's material condition—neither practice retains an association with mentions of dilapidation once post length and platform are controlled (sketching practice OR = 1.12, *p* = 0.795; photography OR = 1.05, *p* = 0.863), and the art-infrastructure feature carries none with negative expression either (OR = 1.21, *p* = 0.235).

### 5.3 Embodied cognition and perceived authenticity

We use authenticity in a constructivist and performative sense: not a property a wall or a village possesses or lacks, but a judgment that visitors, residents, artists, merchants and platforms stage in the course of practice. Our findings therefore qualify MacCannell's “staged authenticity” framework, which posits tourists seeking “back stage” reality behind “front stage” performance, with authenticity defined as correspondence between representation and reality (MacCannell, 1973). It is that correspondence premise, rather than any simple front/back dichotomy—MacCannell himself set out six stages between the two poles—that proves inadequate here. It forces a choice between oyster-shell walls that are genuinely old and walls that are renovated props, whereas many incorporate both original Qing Dynasty shells and recent replacements, and others adopt “heritage style” outright. Most visitors neither know nor care, though those with the longest involvement are a conspicuous exception; their satisfaction derives less from verification of historical accuracy than from embodied engagement in which a meaningful self-experience takes shape, what Wang terms “existential authenticity” (Wang, 2017).

We extend Wang's insights through an embodied cognition framework. For Varela et al. (1991) and Wilson (2002), cognition emerges through bodily interaction with the environment rather than being confined to the brain: perception and meaning co-emerge through practice, sketching producing the tourist-as-artist, photographing the tourist-as-connoisseur, wandering the alleys the tourist-as-explorer. As the sentiment contrasts above show, two practices carried out before the same walls are associated with two different accounts of what the place is: perceived authenticity is neither objective property nor subjective projection but an emergent cognitive achievement.

This reframes heritage tourism's central problematic: rather than asking “Is Xiaozhou authentic?” we ask how Xiaozhou authenticates. Authentication operates less through correspondence to origins than through intensive engagement in which genuineness comes to be felt, and the features carrying it are features of encounter rather than of provenance. Who performs authenticity, and for whom, matters too: negative posts are almost absent from merchant accounts and most frequent among residents, so much of the positive affect on these platforms is supplied by actors with a commercial stake in the village's image. This supports rather than weakens a performative reading, but limits how far platform sentiment can stand in for the community's experience, a limit we return to in Study Limitations and Future Research.

### 5.4 Implications for environmental psychology and place attachment

Place attachment research has traditionally emphasized two dimensions, place dependence and place identity—functional bonds formed through goal-directed activity and emotional-symbolic bonds of self-definition (Williams and Vaske, 2003). Our results suggest that this structure may not cover involvement constituted through sustained practice, and we state that as a limitation of the existing instrument rather than as a new dimension: those scales were validated on survey responses at recreation sites, whereas we work with published text. What they do specify is a valence, and not the one commonly assumed. Negative accounts rise monotonically with the depth of engagement recorded in the text: 10.7% among those passing through or reposting (*n* = 1,797), 22.8% among those reporting a single dedicated visit (*n* = 679), and 28.6% among those reporting repeated visits or extended stays (*n* = 210) [χ^2^(2, *N* = 2,686) = 88.43, *p* < 0.00001]. The highest negative rate of any identifiable group belongs to local residents (33.3%, *n* = 72), whose attachment is the least optional of all. The pattern is not uniform: established artists (*n* = 46) sit at the opposite end, with a mean sentiment of 0.301—higher than that of general visitors and behind only the two commercially interested groups. What separates them is not depth of attachment but position—most hold premises in the village—so that practice-mediated attachment turns critical where the practice depends on conditions the practitioner does not control, and quiet where the practitioner has a stake in how the place is seen. Recreational specialization research has reported this since Bryan (1977): more specialized participants judge a resource's condition more severely than casual users, and Albrecht et al. (2007) named the affective form it takes solastalgia—the distress of those who have not left, but whose place has changed around them. Long-term art students and resident painters write in a consistent “then vs. now” structure, closing accounts of what the village once afforded with the statement that the conditions for their practice are gone. On this evidence, attachment does not buffer environmental criticism; it is a condition of voicing it.

These dynamics are not peculiar to one village. Xiaozhou exemplifies a heritage typology widespread across the Global South yet understudied in Anglophone scholarship: informal settlements that lack the preconditions conventional heritage paradigms assume—bounded zones, enforcement capacity, stable governance—yet sustain vibrant cultural practices (Roy, 2005; Zhang, 2025), and that house a large and growing share of the world's urban population (UN-Habitat, 2024).

One governance implication follows from the results directly. The folding away of environmental deficits is observed at the shallow end of engagement, where visual consumption predominates, and not among those most invested in the village, whose accounts are the most negative overall—though not, as §4.3 reports, the most likely to name a specific condition: the deepest group writes about deterioration in general terms more often than it itemizes it. Supporting art therefore remains cultural policy, but it cannot stand in for environmental maintenance: experiential adaptation buffers perception only up to the point at which material conditions make the practice itself impossible, and the accounts of painters who have left describe precisely that point.

### 5.5 Psychological wellbeing and adaptive environmental engagement

This bears on how sustainability is defined for such places. Heritage tourism scholarship has oscillated between ecological frameworks and socio-cultural concerns, both presuming that sustainability means maintaining current conditions (Gössling and Mei, 2025); our findings suggest instead that it is better understood as adaptive capacity, and also mark where that reconceptualization stops. For much of the visiting population the material fabric deteriorates while reported experience remains positive; among the most invested visitors the two move together rather than apart. Psychological sustainability therefore requires maintaining not buildings as such but the environmental affordances through which embodied practice remains possible—affordances that are themselves material, which is why the most deeply engaged visitors are better read as an early indicator than a minority to be averaged away.

### 5.6 Study limitations and future research

A first constraint is that the corpus records only what people chose to publish. Residents and visitors who do not post, and any experience difficult to render in the format of a platform, are absent, and nothing in the design lets us estimate how large or how different that population is. This bears on the gradient we report: those whose involvement is deepest are also, plausibly, the ones with the strongest reasons both to write and to stay silent, and we cannot separate the two. The governance implications drawn in §6.2 are accordingly offered as hypotheses to be tested against resident-facing methods rather than as findings that speak for the resident population.

A second constraint is that depth of engagement is inferred from the text rather than reported by the author. What we code is the depth a post makes legible—a mention of having studied here, of returning each spring, of what the lane looked like a decade ago—which is not the same as the person's actual involvement. Authors who are deeply involved but write briefly are coded shallow; that error should attenuate the gradient rather than create it, but it also means the three levels are better read as three registers of writing than as three populations of visitors. Two independently developed models agree on the code substantially but not perfectly (κ = 0.695), so a portion of the variance in the gradient is measurement noise in the code itself.

A third constraint concerns what these platforms are for. Users publish what is visually attractive and socially presentable, so that some registers of experience are over-supplied and others withheld. Our own data show this: negative posts make up 1.0% of posts by identifiable merchants (*n* = 192) and 7.5% of posts by media and marketing accounts, against 33.3% of posts by identifiable residents (*n* = 72), whose mean sentiment (0.048) is the lowest of any identifiable group. Sentiment distributions derived this way describe a public and partly promotional discourse rather than what visitors and residents privately feel, and will not align straightforwardly with results obtained through interviews or surveys.

Regarding causal inference, SHAP values quantify each feature's marginal contribution to the model's predictions; they do not measure effects in physical space, and we advance no causal claim on their basis. The design is cross-sectional and observational, so directionality cannot be established. We cannot rule out reverse causation—visitors already disposed to notice decline may be the ones who take up sustained practice—nor confounding by the period a post refers to, since deeply engaged authors write about a longer stretch of the village's history than one-time visitors do. The association between depth of engagement and expressed negativity is a robust statistical pattern in how practices, elements and affective responses co-vary in text, not evidence of causal efficacy. The models set a further limit on any single attribution: the gradient-boosting specification reaches *R*^2^ = 0.137 under ten-fold cross-validation (RMSE 0.635, MAE 0.495; with the annotation score as dependent variable, 0.172), so 18 transparent lexicon features account for a modest share of the variation.

Temporally, our corpus spans 2008–2026, with coverage thickening from 2018 onwards (more than 100 records per year thereafter), so that it captures Xiaozhou's intensive touristification while the earliest phase of its artistic colonization and its pre-transformation baseline enter only through retrospective accounts written later; longitudinal analysis tracking the full lifecycle would illuminate tipping points more fully. As a single-case study, our findings transfer theoretically rather than statistically: Xiaozhou's coupling of art, heritage and nature, its informal governance and its peri-urban location may generate dynamics differing from those of other heritage typologies.

A further limitation concerns the unit of analysis: the models are fitted at the level of the record—a post or a comment—while the argument is about people. A single author may contribute several posts, and the platforms expose no stable author identifiers that would let us group them; the 9,643 comments are moreover nested within the 2,850 posts they respond to, so comment-level observations are not independent in the way a flat model assumes. We have not corrected for either kind of clustering, so the confidence intervals reported here should be read as narrower than they would be under a multilevel specification. The direction and rough magnitude of the contrasts hold separately at the post level, but the precision does not.

A further caution concerns who writes at each level. Promotional accounts concentrate among the least engaged posts, and the intermediate step is not separable from unity once author position is controlled (§4.3). The contrast that carries the argument holds in every specification, but the subgroup analyses rest on as few as 180 negative cases; a design sampling authors rather than posts would estimate it more precisely.

Two predictions follow from this reading, and both can fail. The first is comparative: if the direction of folding is set by depth of involvement rather than by the activity pursued, then at heritage sites that do not support sustained creative presence—purely sightseeing-oriented old towns, sites with no tradition of visitors working there—the rise in negative expression across levels of engagement depth should be markedly flatter, or absent. Should the gradient instead appear wherever it is looked for, the more likely explanation is that longer involvement simply produces longer and more differentiated writing, and the account offered here would be wrong. The second prediction is internal: if depth rather than medium is what matters, then photographers whose involvement is deep—those on long residencies, or locals who have photographed the same lanes for years—should express negativity at rates approaching those of long-dwelling painters rather than remaining at the low rate we observe. Should they remain low at every depth, what is doing the work is the selectivity of the medium rather than the depth of the involvement, and the argument would have to be restated in those terms.

The inference at the center of our argument calls for instruments this design did not have: surveys and interviews measuring recreational specialization on validated scales (Bryan, 1977) and place attachment on established ones (Williams and Vaske, 2003), administered to residents, art students, resident artists and one-time visitors alike, would show whether the gradient we observe in published text also appears when people are asked directly—and would reach the people who never post.

## 6 Conclusion

### 6.1 Key empirical findings and contributions

This study set out to resolve a paradox: how living heritage sites sustain perceived cultural authenticity despite visible material degradation. Analyzing Xiaozhou Village—a rapidly urbanizing water settlement in Guangzhou combining severe environmental decline with robust visitor satisfaction—we have argued that heritage resilience is associated not with static preservation but with what we term “topological folding”: the affective-spatial reconfiguration whereby some elements achieve experiential proximity while others recede to topological distance. The direction of that folding is not fixed; it is contingent on the depth of a visitor's embodied engagement.

Across 12,493 records−2,850 posts and 9,643 comments from 13 Chinese platforms—none of the three largest positive contributors to predicted sentiment is an architectural feature: affective experience is associated less with material attributes than with the relations between practices, elements and visitors, a weighting difficult to reconcile with paradigms privileging material authenticity (Smith, 2006; Winter, 2014).

Two findings organize the account. First, what conditions attention to environmental decline is depth of involvement rather than the activity pursued: the share of negative posts climbs from 10.7% among those passing through, to 22.8% among those describing a dedicated visit, to 28.6% among those who return repeatedly or stay [χ^2^(2, *N* = 2,686) = 88.43, *p* < 0.00001], and the deepest contrast survives every adjustment we applied, including one for who is writing (OR = 3.29 before and 2.14 after; §4.3). The intermediate level depends on author composition, so the result is stated as a contrast between sustained involvement and passing contact rather than as a strictly monotonic gradient. No single activity accounts for it, and an earlier lexicon that appeared to show one had conflated painting with the studios and academies that host it (§4.3). Second, the same ordering appears across author positions: residents record the highest share of negative posts (33.3%) and commercial accounts the lowest (1.0%), with established artists a conspicuous exception at 13.0%.

Theoretically, what the data specify is an ordering rather than a threshold ratio: depth of embodied engagement is the variable along which heterogeneous elements are differently configured. Recreation specialization theory (Bryan, 1977), developed for anglers, anticipates the evaluative severity we observe at the specialized end of that continuum; and what we recover there, an insistence on what the place used to be from people who remain attached to it, is better described as solastalgia (Albrecht et al., 2007) than as dissatisfaction with a destination. Our findings therefore qualify a widely held expectation in place attachment research—though not any published claim, since the framework we build on (Williams and Vaske, 2003) takes no position on valence: stronger attachment is associated with more critical evaluation, because attachment is part of what makes deterioration legible.

Methodologically, explainable AI is used here as a theory-generative method rather than as predictive validation, and the claim is narrow: cross-model agreement is evidence of reliability, transparent lexicon features of auditability, and neither establishes construct validity. Auditability is what makes validity testable, and here that was decisive—tracing an anomalous result back to its words showed a feature named for an activity being triggered by the institutions surrounding it (§4.3), and correcting the measure removed the result.

### 6.2 Practical implications and governance recommendations

Two implications follow directly. Aggregate satisfaction is the last indicator to move: the same corpus that is broadly positive overall returns 28.6% negative posts among those who return repeatedly or stay and 33.3% among identifiable residents, so monitoring that averages across all visitors registers decline only after the people who sustain its cultural production have begun to leave. Protecting practices, in turn, means protecting the material conditions they require—air, quiet, affordable rent—which are what practitioners report losing, several accounts tracing the studios' departure to rents that rose with the visitor numbers (lifestyle-sharing platform, 2026). Experiential sustainability does not substitute for environmental sustainability: governance built on adaptive capacity has to keep material and ecological remediation inside it.

### 6.3 Closing reflection

This study set out to ask what conditions the way environmental conditions enter visitors' accounts of a heritage place, and it returns three answers. Visitors do reorganize environmental attention, and the reorganization is legible in what they write: the same canal appears as Lingnan water-town form in one account and as turbid water in another, and the difference does not lie in the canal. Deeper involvement does not attenuate negative environmental cues but heightens sensitivity to them—the share of negative accounts rises across the three levels of engagement, and the contrast between those most involved and those passing through survives adjustment for how much each group writes, for platform, for type of activity and for who is writing. And what conditions that direction is the depth and position of involvement rather than the kind of activity pursued: sketching, the activity most closely identified with Xiaozhou's reputation, shows no detectable association with negative expression once depth is held constant, while those who return repeatedly or stay are the readiest to name what is failing.

This runs against the convergent prediction of processing fluency, effort justification and, on its usual reading, place attachment, and with recreation specialization and solastalgia. Attachment appears to supply not only a reason to value a place but a standard against which to judge it, together with a stake in whether the place meets that standard. For environmental psychology the consequence is that the fold is located not in the site but in the biography of engagement brought to it; for heritage management it is that the most critical voices in a corpus are likely to belong to those most invested in the place, and are better read as a form of attachment than as hostility to it.

Xiaozhou Village points to what preservation orthodoxy obscures: cultural vitality emerges from transformation rather than stasis, from relation rather than essence, from capacity rather than control. The oyster-shell walls matter less because they embody historical authenticity than because they are enrolled in artistic assemblages within which meaningful experience takes shape—up to the point at which the practice becomes impossible, a point the village's painters report having reached.

For the thousands of informal heritage settlements without UNESCO designation or preservation resources, yet sustaining cultural practices through vernacular adaptation, the question is no longer “How do we prevent change?” but “How do we sustain cultural meaningfulness through inevitable transformation, and for whom?” Xiaozhou Village offers a partial answer and a warning in the same breath: its assemblage does fold degradation to the experiential periphery for many who encounter it, but the folding is neither free nor uniform. Those most engaged are the ones for whom degradation folds closest, and they are also the ones who can leave.

Heritage, properly understood, is not what we preserve from the past but what we continuously assemble in the present.

## Data Availability

The original contributions presented in the study are included in the article/Supplementary material, further inquiries can be directed to the corresponding author.
